# First-line sintilimab plus chemotherapy in locally advanced or metastatic esophageal squamous cell carcinoma: A cost-effectiveness analysis from China

**DOI:** 10.3389/fphar.2022.967182

**Published:** 2022-12-07

**Authors:** Jian Shen, Yi Du, Rong Shao, Rong Jiang

**Affiliations:** ^1^ Institute of Regulatory Science, China Pharmaceutical University, Nanjing, Jiangsu, China; ^2^ NMPA Key Laboratory for Drug Regulatory Innovation and Evaluation, China Pharmaceutical University, Nanjing, Jiangsu, China

**Keywords:** sintilimab, chemotherapy, esophageal squamous cell carcinoma, cost-effectiveness analysis, QALYs, ICER, incremental cost-effectiveness ratio

## Abstract

**Objective:** The study aimed to assess the cost-effectiveness of sintilimab combined with cisplatin plus paclitaxel versus chemotherapy alone as first-line treatment in patients with advanced or metastatic esophageal squamous cell carcinoma from the Chinese healthcare system.

**Materials and methods:** A partitioned survival model was developed based on the ORIENT-15 clinical trial. Drug costs and health state utility were obtained from the literature. Outcomes included the health outcomes in life-years, quality-adjusted life-years (QALYs), and the incremental cost-effectiveness ratio. One-way and probabilistic sensitivity analyses were performed to evaluate the model uncertainty.

**Result:** In overall population, patients given sintilimab plus chemotherapy gained more health benefits (0.90 QALYs vs. 0.61 QALYs), and the cost was more (15,399.21 US$ VS. 7475.58 US$) than that for patients in the chemotherapy group. In the subgroup, patients given sintilimab plus chemotherapy gained more health benefits (0.89 QALYs vs. 0.68 QALYs), and the cost was more (15,656.19 US$ vs. 9,162.77 US$) than that for patients in the chemotherapy group. Compared with chemotherapy, patients receiving sintilimab plus chemotherapy had ICERs of $26,773.68/QALY in the overall population and $30,065.50/QALY in the subgroup, which was above the threshold of WTP.

**Conclusion:** Sintilimab plus chemotherapy was more cost-effective than chemotherapy alone for patients with advanced esophageal cancer from the perspective of the Chinese healthcare system.

## Introduction

Esophageal cancer is the tenth most common cancer and the sixth most frequent cause of tumor-related deaths. There are approximately 600,000 new cases of esophageal cancer and more than 500,000 deaths worldwide each year (Sung et al., 2021). Esophageal squamous cell carcinoma is the predominant subtype of esophageal cancer in the Asian population, occurring in 90% of patients (Bray et al., 2018; Arnold et al., 2020), especially for China. It was estimated that 477,900 people in China would be diagnosed with esophageal cancer, 90% of which was histologically identified as squamous cell carcinoma, and 375,000 of these patients would die from the disease (Chen et al., 2016; Li et al., 2017).

Platinum-based chemotherapy is the common first-line treatment for patients with advanced or metastatic esophageal squamous cell carcinoma in China. Patients usually received platinum plus paclitaxel, while platinum plus 5-fluorouracil is preferred in other countries. According to METGastric (NCT01662869) and POWER (NCT01627379), the median overall survival is less than 12 months, which is the minimal benefit for patients with esophageal cancer treated with these regimens (Shah et al., 2017; Moehler et al., 2020).

Sintilimab, a fully recombinant human IgG4 anti-PD-1 monoclonal antibody, has been approved for the treatment of classical Hodgkin’s lymphoma, non-small cell lung cancer, and hepatocellular carcinoma by the National Medical Products Administration of China (Yang et al., 2020; Ren et al., 2021). The ongoing ORIENT-15 (a multicenter, double-blinded, randomized, phase-3 clinical trial of sintilimab in combination with chemotherapy versus chemotherapy only; [ClinicalTrials.gov, identifier: NCT03748134]) found that compared with cisplatin plus paclitaxel, sintilimab in combination with cisplatin plus paclitaxel showed significant benefits in overall survival and progression-free survival as the first-line treatment in patients with advanced or metastatic esophageal squamous cell carcinoma. Similar benefits of sintilimab with cisplatin plus 5-fluorouracil seem promising (Lu et al., 2022).

Despite the efficacy of sintilimab plus chemotherapy for the first-line treatment of advanced or metastatic esophageal squamous cell carcinoma, one must consider the high costs of the agent. These high costs can cause financial toxicity for patients, leading patients to delay or forgo the treatment, decreasing the quality of life, and even putting patients at the risk of bankruptcy (Abbott et al., 2017; Desai and Gyawali, 2020). In addition to that, advanced esophageal squamous cell carcinoma has a high incidence rate in China, and the adoption of costly drugs could add to the increasing costs of cancer care in general. These economic healthcare concerns suggest the assessment of the value or cost-effectiveness of these drugs is needed for Chinese patients. The aim of this study was to evaluate the cost-effectiveness of sintilimab plus chemotherapy compared with chemotherapy alone as first-line treatment for Chinese patients with locally advanced or metastatic esophageal squamous cell carcinoma from the perspective of the Chinese healthcare system based on the ORIENT-15 trial data.

## Materials and methods

### Patient population and intervention

Patient population and intervention were based on the patients in the ORIENT-15 trial. Target patients were with histologically confirmed unresectable, locally advanced, recurrent, or metastatic esophageal squamous cell carcinoma; were unsuitable for curative intent surgery or definite concomitant chemoradiotherapy; had received no previous systemic treatment (patients who had progressed >6 months after adjuvant or neoadjuvant chemotherapy or definitive chemoradiotherapy were eligible); could provide a fresh or archival tumor sample to evaluate expression of PD-L1; had at least one measurable lesion, based on the Response Evaluation Criteria in Solid Tumors (RECIST), version 1.1, assessed by the investigators; had an Eastern Cooperative Oncology Group (ECOG) performance status score of 0 or 1; and had adequate hematological organ function. Key exclusion criteria were tumor invasion in the aorta or trachea, hepatic metastasis of >50% of the total volume of the liver, a diagnosis of other malignant tumors, active autoimmune disease or a history of active autoimmune disease, and interstitial lung disease requiring corticosteroids. The ORIENT-15 trial made a subgroup by stratified overall population into those with the expression of the PD-L1 tumor proportion score ≥10%.

Baseline characteristics were similar in the two groups (Table 1). Of 659 patients included, 640 (97%) were from 66 sites in China, and 19 (3%) were from 13 sites outside of China. All enrolled patients had esophageal squamous cell carcinoma; 57% (188/327) and 58% (193/332) of patients had PD-L1 CPS ≥10 in the sintilimab plus chemotherapy group and the chemotherapy group, respectively. Overall, 93% (616/659) of patients received cisplatin plus paclitaxel (Table 1).

**TABLE 1:** Baseline characteristics of the patients.

	Sintilimab plus chemotherapy	Standard chemotherapy
Age (years)		
Median (IQR)	63 (57–67)	63 (57–67)
<65	189 (58%)	202 (61%)
≥65	138 (42%)	130 (39%)
Gender		
Men	279 (85%)	288 (87%)
Women	48 (15%)	44 (13%)
Race		
Asian	320 (98%)	321 (97%)
White	4 (1%)	8 (2%)
Not reported	3 (1%)	3 (1%)
Disease status at enrollment		
Metastatic	285 (87%)	287 (86%)
Local advanced	42 (13%)	45 (14%)
Chemotherapy regimen		
Cisplatin plus paclitaxel	307 (94%)	309 (93%)
Cisplatin plus 5-fluorouracil	20 (6%)	23 (7%)
PD-L1 expression (CPS)		
CPS <10	139 (43%)	139 (42%)
CPS ≥10	188 (57%)	193 (58%)

Abbreviation: IQR, interquartile range; CPS, combined positive score

As for the intervention, every 3 weeks, all patients received sintilimab combined with chemotherapy or standard chemotherapy. The chemotherapy regimen was chosen by the investigator: cisplatin plus paclitaxel or cisplatin plus 5-fluorouracil. Sintilimab was given intravenously at a dose of 200 mg in patients weighing ≥60 kg on day 1 of each cycle. Cisplatin (75 mg/m^2^ on day 1 of each cycle) plus paclitaxel (87.5 mg/m^2^ on day 1 and day 8 of cycle 1; 175 mg/m^2^ on day 1 of the other cycles) or 5-fluorouracil (800 mg/m^2^ continuous administration on days 1–5 of each cycle) was also given intravenously.

We assumed that patients would receive docetaxel alone and best support care as subsequent treatment. In the sintilimab plus chemotherapy and chemotherapy-alone groups, 41% and 54% of individuals, respectively, received subsequent chemotherapy (Ajani et al., 1994; National Comprehensive Cancer Network, 2021; Lu et al., 2022). We assumed a typical patient who weighed 65 kg and had a height of 1.64 m (body surface area of 1.72 m^2^) in order to calculate the dosage of sintilimab and chemotherapy agents (Zhang et al., 2020).

### Model overview

**FIGURE 1:**
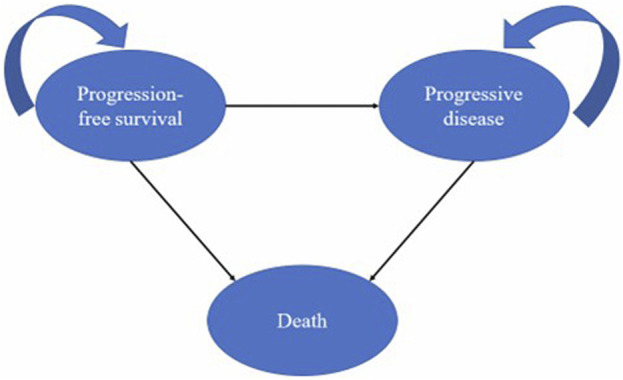
Model structure.

The study established a three-state partitioned survival model in Microsoft Excel 2019 with mutually exclusive health states of progression-free survival (PFS), progressive disease (PD), and death (Figure 1). We assume all patients started with the PFS state. When patients entered the model from the PFS state, they could survive the PFS state, enter the PD state, or the death state. All patients who were transferred from PFS to PD could not recover from their PFS state but continued to progress or die.

To facilitate parameter calculation, we set the duration of the cycle of the partitioned survival model to 3 weeks. The time horizon was the whole life time, which ended when 99% patients in the cohort did not survive and died. In addition, considering the current economic development of China, we used a 5% discount rate. In this model, life-time healthcare costs, quality-adjusted life-years (QALYs), and incremental cost-effectiveness ratios (ICERs) were primary outputs.

### Clinical input

The most available data for sintilimab combination with chemotherapy and standard chemotherapy overall survival (OS) and PFS were derived from the ORIENT-15 study (Lu et al., 2022). The Kaplan–Meier (KM) curves from ORIENT-15 study trials were converted to numeric values through digitalization using the software application “GetData Graph Digitizer”.

**FIGURE 2:**
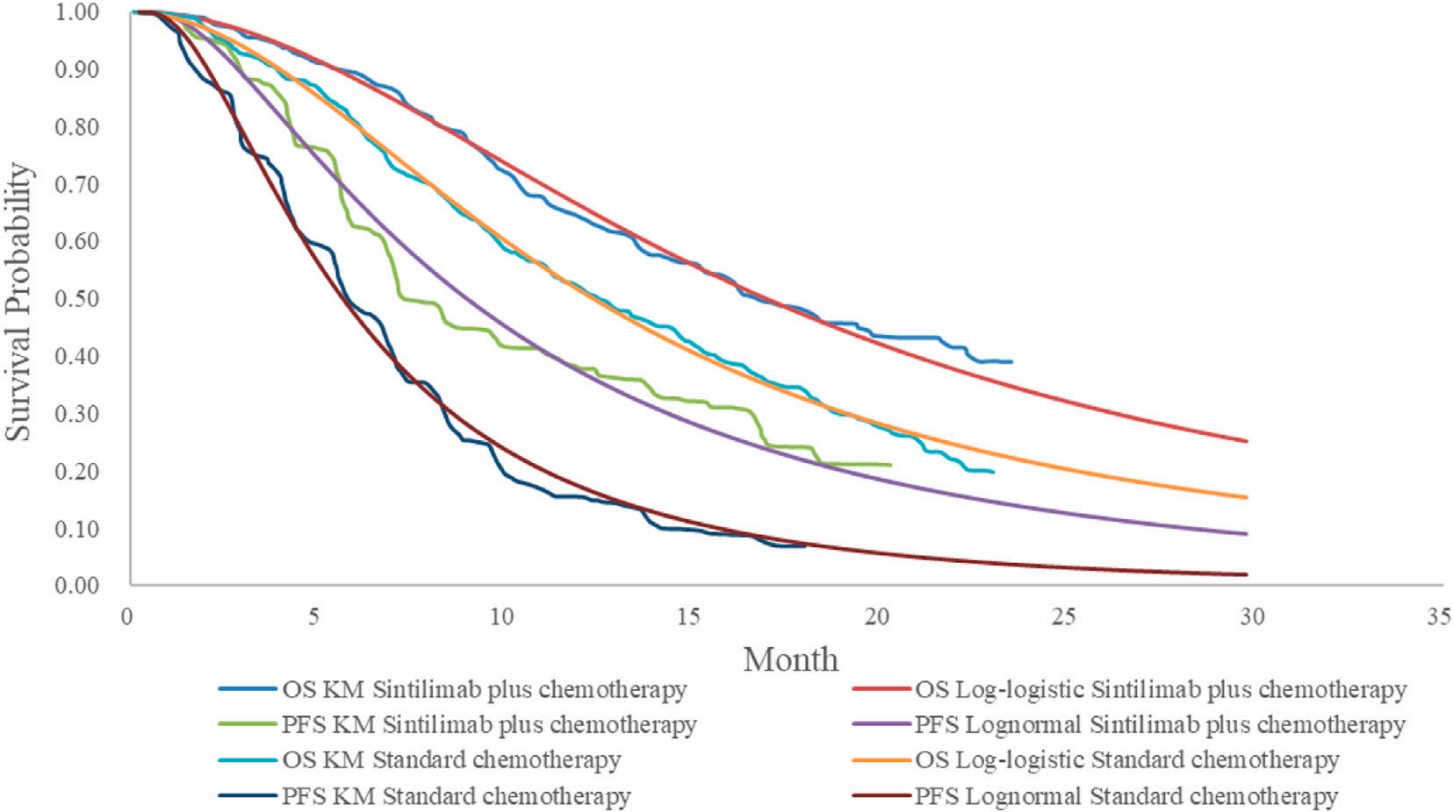
Estimated survival curves for progression-free survival and overall survival in all patients derived from the ORIENT-15 trial.

**FIGURE 3:**
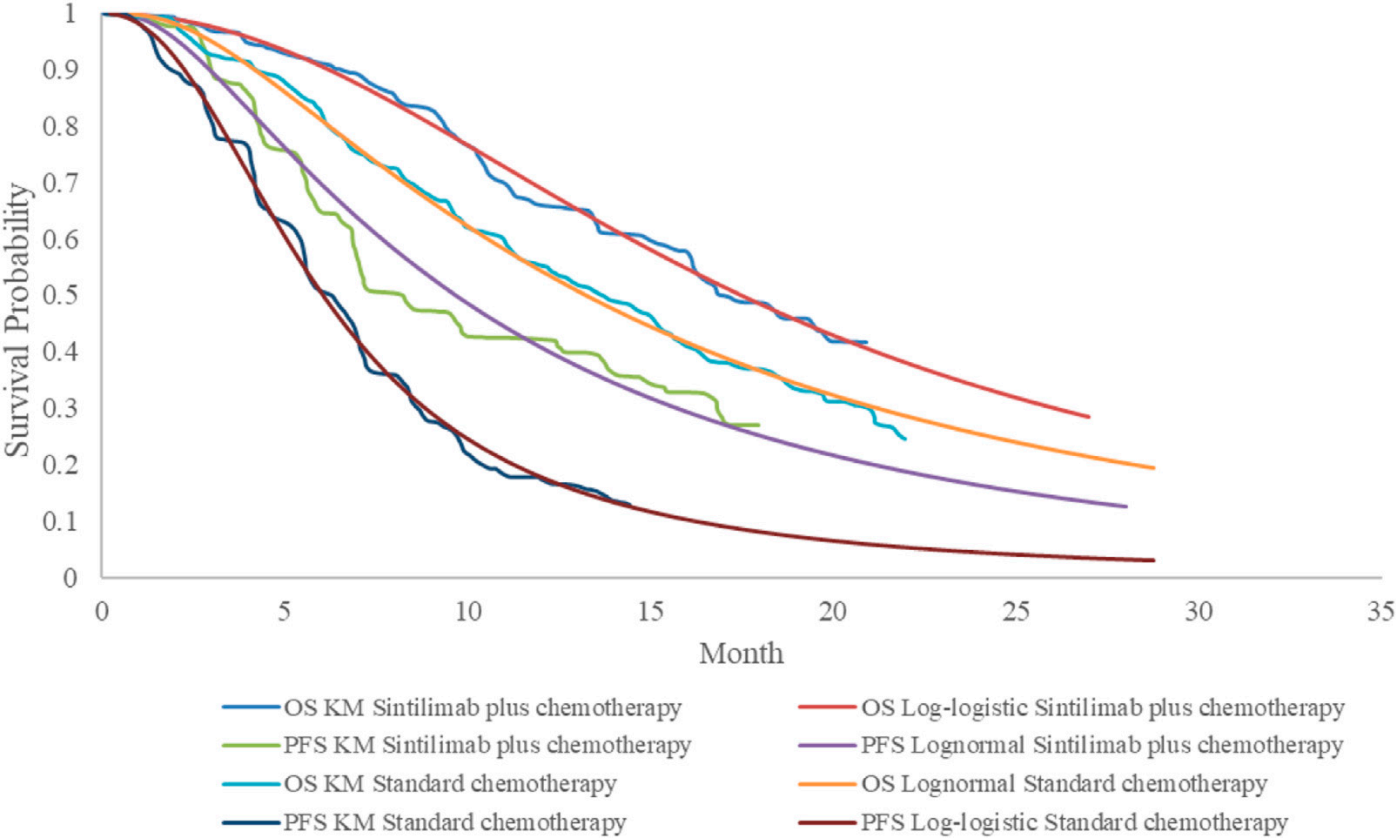
Estimated survival curves for progression-free survival and overall survival in patients with CPS ≥10 derived from the ORIENT-15 trial.

We selected the survival curves with the best fit for PFS and OS, respectively, based on the Akaike and Bayesian information criteria (AIC and BIC) (Table 2) and visual inspection (Figures 2, 3). The best fit distributions and relative parameters for all survival curves can be found in Table 3.

**TABLE 2:** Value of AIC and BIC.

Type of distribution	Sintilimab plus chemotherapy OS		Sintilimab plus chemotherapy PFS		Sintilimab plus chemotherapy PFS		Standard chemotherapy OS	
	AIC	BIC	AIC	BIC	AIC	BIC	AIC	BIC
In all patients								
Exponential	649.7547	653.5446	726.1925	729.9825	758.885	762.6901	752.2067	756.0119
Weibull	615.6429	623.2228	698.0775	705.6574	718.0952	725.7055	692.5922	700.2025
Gompertz	631.4136	638.9936	720.9019	728.4818	735.6034	743.2137	725.2064	732.8166
Log-normal	613.2866	620.8665	672.4213	680.0012	714.9304	722.5407	679.4262	687.0364
Log-logistic	610.3871	617.967	675.2949	682.8749	713.626	721.2362	680.4558	688.0661
Gen-gamma	613.2811	624.651	673.661	685.0308	714.4419	725.8573	679.9881	691.4035
In patients with CPS ≥ 10								
Exponential	359.0264	362.2628	498.8872	502.1236	430.3089	433.5716	425.3247	428.5874
Weibull	331.9929	338.4658	487.4581	493.931	407.0693	413.5947	388.1698	394.6952
Gompertz	340.1515	346.6244	498.7202	505.1931	416.9549	423.4803	405.877	412.4023
Log-normal	333.4613	339.9342	468.1432	474.6161	403.9922	410.5176	382.7506	389.276
Log-logistic	330.6123	337.0852	471.3219	477.7948	404.7849	411.3103	382.408	388.9334
Gen-gamma	333.0212	342.7305	469.1594	478.8687	405.4148	415.2029	383.6583	393.4464

Abbreviation: OS, overall survival; PFS, progression-free survival; AIC, Akaike information criterion; BIC, Bayesian information criterion; CPS ≥ 10, combined positive scores ≥10.

**TABLE 3:** Shape parameter and scale parameter.

OS and PFS curve	Optimal fitting distribution	Shape parameter	Scale parameter
In overall population			
OS curves of sintilimab plus chemotherapy	Log-logistic	2.828918	0.5166103
PFS curves of sintilimab plus chemotherapy	Lognormal	2.1923	0.8939621
OS curves of chemotherapy	Log-logistic	2.512269	0.513398
PFS curves of chemotherapy	Lognormal	1.733157	0.7921379
In patients with CPS ≥ 10			
OS curves of sintilimab plus chemotherapy	Log-logistic	2.862995	0.4700579
PFS curves of sintilimab plus chemotherapy	Lognormal	2.270911	0.9233276
OS curves of chemotherapy	Lognormal	2.583066	0.8959587
PFS curves of chemotherapy	Log-logistic	1.800978	0.4471674

Abbreviation: OS, overall survival; PFS, progression-free survival

### Quality-of-life inputs

We integrated health-related quality of life into the model by using utility values. Utility values are based on personal preferences and reflect the degree of health status people expect. Typically, the value of death is 0, and the value of a fully healthy individual is 1, while a patient’s health status is usually between 0 and 1. The utility values used in this analysis were derived from the published related literature (Table 4). The utility values of PFS and PD health statuses in our model were 0.741. and 0.581, respectively (Wu et al., 2012; Zhang et al., 2020). An annual discount rate of 5% was applied to all QALYs.

**TABLE 4:** Key inputs for the cost-effectiveness model.

Parameter	Value	Low	High	Distribution	Source
Cost (US$)					
Sintilimab/1000 mg	167.40	133.92	200.88	Gamma	Negotiation price
Cisplatin/50 mg	11.78	9.42	14.14	Gamma	Local charge
Paclitaxel/30 mg	11.44	9.15	13.73	Gamma	Local charge
5-Fluorouracil/250 mg	9.61	7.69	11.53	Gamma	Local charge
Docetaxel/20 mg	27.90	22.32	33.48	Gamma	Local charge
Cost of BSC	117	32.3	322.6	Gamma	Zhang et al. (2020)
Cost of the follow-up	52.80	36.96	68.64	Gamma	Shi et al. (2021)
Chest enhanced CT	134.37	107.50	161.24	Gamma	Shi et al. (2021)
Abdominal enhanced CT	134.37	107.50	161.24	Gamma	Du et al. (2022)
Cost of the laboratory test	90.32	72.26	108.38	Gamma	Meng-xue et al. (2022)
Other cost					
Anti-tumor drug dispensing fees	4.01				
Cost of IV	2.78				
Hospitalization expense/day	19.86				
Cost of SAEs					
Anemia	669.45	535.56	803.34	Gamma	Du et al. (2022)
Decrease in the white blood cell count	206.72	165.376	248.06	Gamma	Du et al. (2022)
Decrease in the neutrophil count	544.19	435.35	613.43	Gamma	Du et al. (2022)
Asthenia	115.00	92.00	138.00	Gamma	Yang et al. (2021)
Increase in blood pressure	1.35	1.08	1.62	Gamma	Wen et al. (2021)
Utility					
PFS	0.741	0.593	0.889	Beta	Wu et al. (2012); Zhang et al. (2020)
PD	0.581	0.465	0.697	Beta	Wu et al. (2012); Zhang et al. (2020)
Disutility due to grade 1 and 2 AEs	0.01	0.008	0.02	Beta	Amdahl et al. (2016); Su et al. (2021)
Disutility due to grade 3 and 4 AEs	0.16	0.11	0.204	Beta	Amdahl et al. (2016); Su et al. (2021)
Accounting rate	5%	0%	8%	Fix	

Abbreviation: BSC, best support care; SAEs, serious adverse events (≥ grade 3); IV, intravenous injection; PFS, progression-free survival; PD, progression disease; CT, computerized tomography

Experiencing adverse events was considered a decrease in health utility otherwise known as a disutility. We considered the disutility values due to grade 1 or 2 and grade 3 or 4 AEs in order to accurately obtain the result of this analysis (Amdahl et al., 2016; Su et al., 2021). All AEs were assumed to have incurred during the first cycle.

### Cost input

This analysis was conducted from the perspective of the Chinese healthcare system. Therefore, the cost mainly included drug costs, costs of best supportive care (BSC), chemotherapy costs, testing cost, follow-up costs, and treatment costs associated with grades 3 and 4 adverse events. All costs in this study were reported in 2021 US dollars with an exchange rate of US 1 USD = 6.4515 Chinese yuan (National Bureau of Statistics of China, 2022).

The cost of drugs came from the national medical insurance negotiation price and local charge, and other costs were derived from the previously published research studies and related literature. The dosage of the drug taken by the patients was the same as the ORIENT-15 study. Treatment costs per cycle were calculated by using dosing schedules based on the local price (Table 4). Because sintilimab or chemotherapy was given intravenously, administration costs were included too.

Serious adverse event (SAE)-related costs were included as a weighted average based on the number of reported adverse events in the clinical trial. SAE-related cost was calculated by multiplying the incidence rate of SAEs with the cost of managing these adverse events per event. All SAEs were assumed to be incurred during the first cycle.

### Sensitivity analysis

We performed one-way analysis (OSA) and probabilistic sensitivity analysis (PSA) for resolving the uncertainty in the model. In the one-way sensitivity analyses, the range of parameters used was derived from the published literature. When the data range in the literature was unavailable, the baseline value was used to float up and down by 20% (Li et al., 2021). The discount rate in one-way analyses ranged from 0 to 8%. As for the probabilistic sensitivity analysis (PSA), it was conducted by jointly varying all model parameters over 1,000 Monte Carlo simulations in order to evaluate the probability of each intervention being cost-effective, given that different values of WTP for an additional QALY are derived. In addition, for variables used in Monte Carlo simulation, beta distributions were assigned to utility parameters, and gamma distributions were assigned to costs. A scatter plot and a cost-acceptable curve would present the final result.

## Results

### Base-case analysis

The partitioned survival model was established to assess the cost-effectiveness of sintilimab plus chemotherapy and chemotherapy alone in the treatment of advanced or metastatic esophageal squamous cell carcinoma by using QALYs and long-term cost. The important endpoint of the assessment was ICER.

Compared with chemotherapy alone, sintilimab plus chemotherapy yielded increases in QALYs both in overall population and population with PD-L1 CPS ≥10. For the overall population, the total costs of the sintilimab plus chemotherapy group in China were US$ 15,399.21, while the total costs of the chemotherapy group were US$ 7,475.58. The QALYs for the sintilimab plus chemotherapy and chemotherapy-alone groups were 0.90 and 0.61, respectively. Patients treated with sintilimab plus chemotherapy gained an additional 0.30 QALYs. The final ICER in this analysis was US$ 26,773.68. For the population with PD-L1 CPS ≥10, sintilimab plus chemotherapy yielded additional 0.31 LYs and 0.22 QALYs at an incremental cost of US$ 6,493.43, producing an ICER of 30,065.50 US$/QALY (Table 5).

**TABLE 5:** Results for the cost utility analysis.

	Total cost (US$)	LY	QALY	Δ cost (US$)	ΔQALY	ICER (US$/QALY)
Overall population						
Sintilimab plus chemotherapy	15,399.21	1.44	0.90	7,923.63	0.30	26,773.68
Chemotherapy alone	7,475.58	1.04	0.61	NA	NA	NA
Population with PD-L1 CPS ≥ 10						
Sintilimab plus chemotherapy	15,656.19	1.39	0.89	6,493.43	0.22	30,065.50
Chemotherapy alone	9,162.77	1.08	0.68	NA	NA	NA

Abbreviation: QALYs, quality-adjusted life-years; ICER, incremental cost-effectiveness ratio; NA, Not applicable; Note: Δ, incremental

Because currently there was no established willingness-to-pay threshold in Chinese Pharmaco-economic Evaluation Guidelines, this study compared ICER based on the metrics established by the World Health Organization, which was three times the per capita GDP of China in 2021 (National Bureau of Statistics, 2021). According to the WHO, when the ICER was less than the threshold, the increased cost was regarded as acceptable; when the ICER was greater than the threshold, the increased cost was not worthwhile. China’s per capita GDP in 2021 was 12,551.50 US$, so the threshold was 37,654.50 US$/QALY. According to the base-case analysis, the ICERs for the sintilimab plus chemotherapy and chemotherapy-alone groups were 26,773.68 US$/QALY and 30,065.50 US$/QALY, respectively, which were below the specified willing-to-pay threshold 37,654.50 US$.

### Sensitivity analysis

**FIGURE 4:**
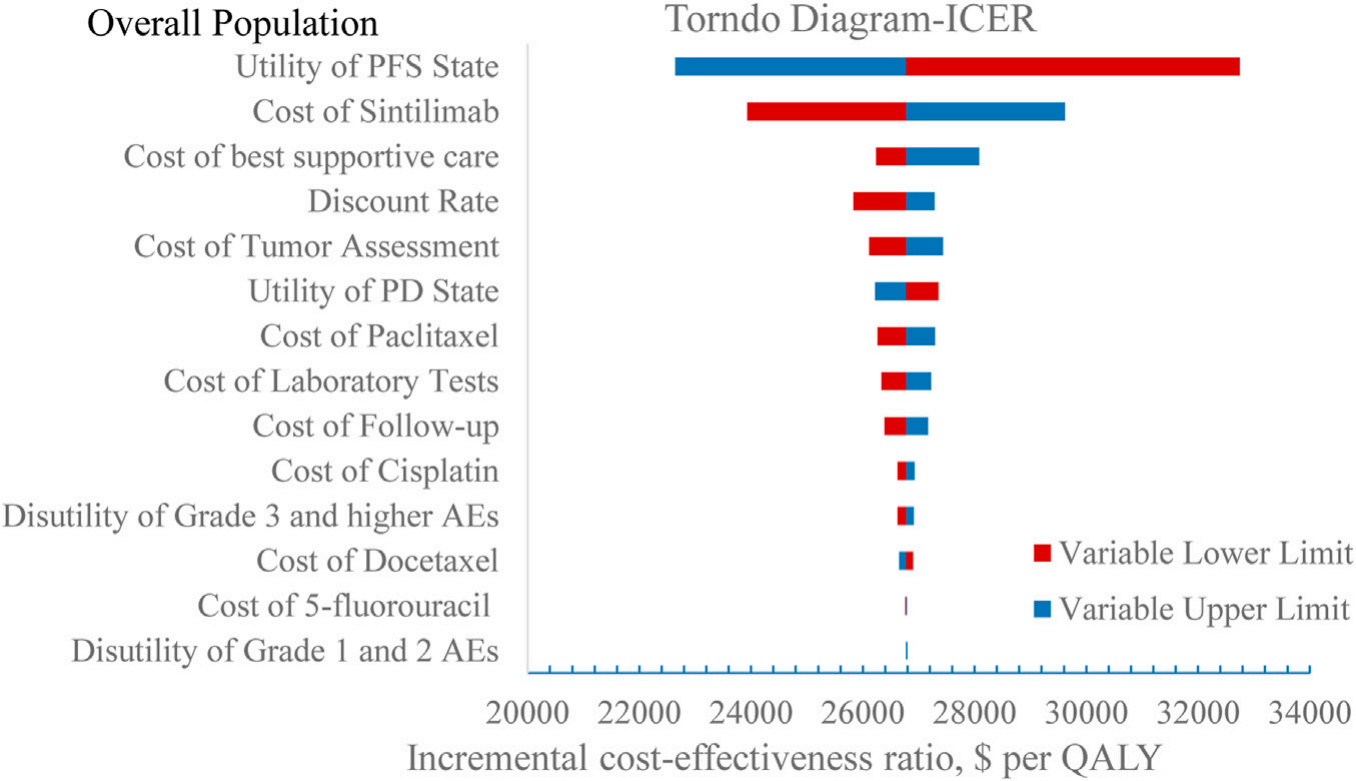
One-way sensitivity analysis in overall population.

**FIGURE 5:**
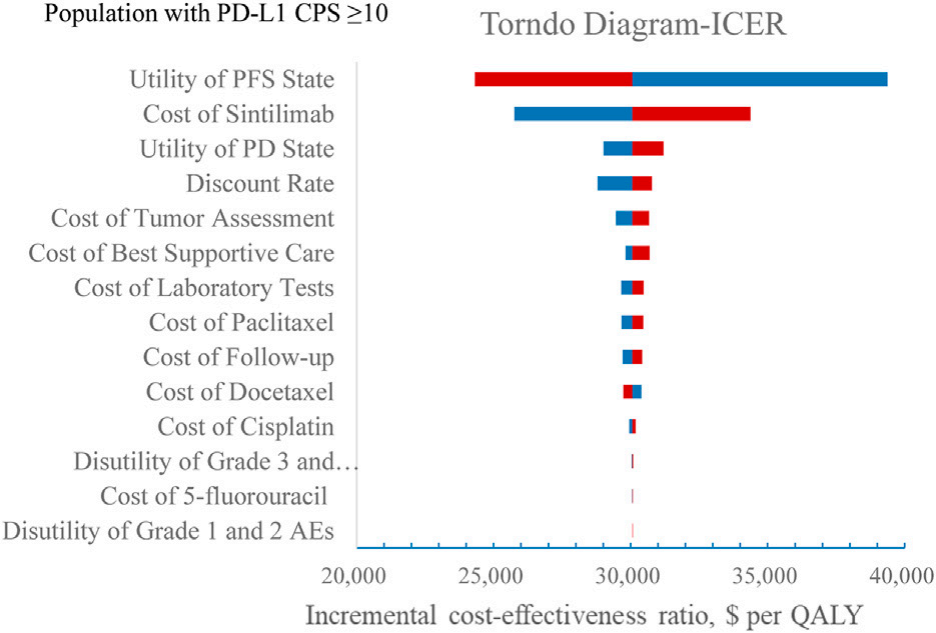
One-way sensitivity analysis in the subgroup. Abbreviation: BSC, best supportive care; PFS, progression-free survival; PD, progression disease; EV, expected value; ICER, incremental cost-effectiveness ratio.

The stability of the results was verified by one-way sensitivity analysis. The tornado diagrams are shown in Figures 4, 5. In the one-way sensitivity analysis, the main model drivers were the utility of the PFS state and the cost of sintilimab both in overall population and population with PD-L1 CPS ≥10. At the highest end of the utility of the PFS state in the one-way sensitivity analysis, the incremental cost-effectiveness ratio (ICER) remained under a 37,654.50 US$/QALY threshold, indicating that our results were robust.

**FIGURE 6:**
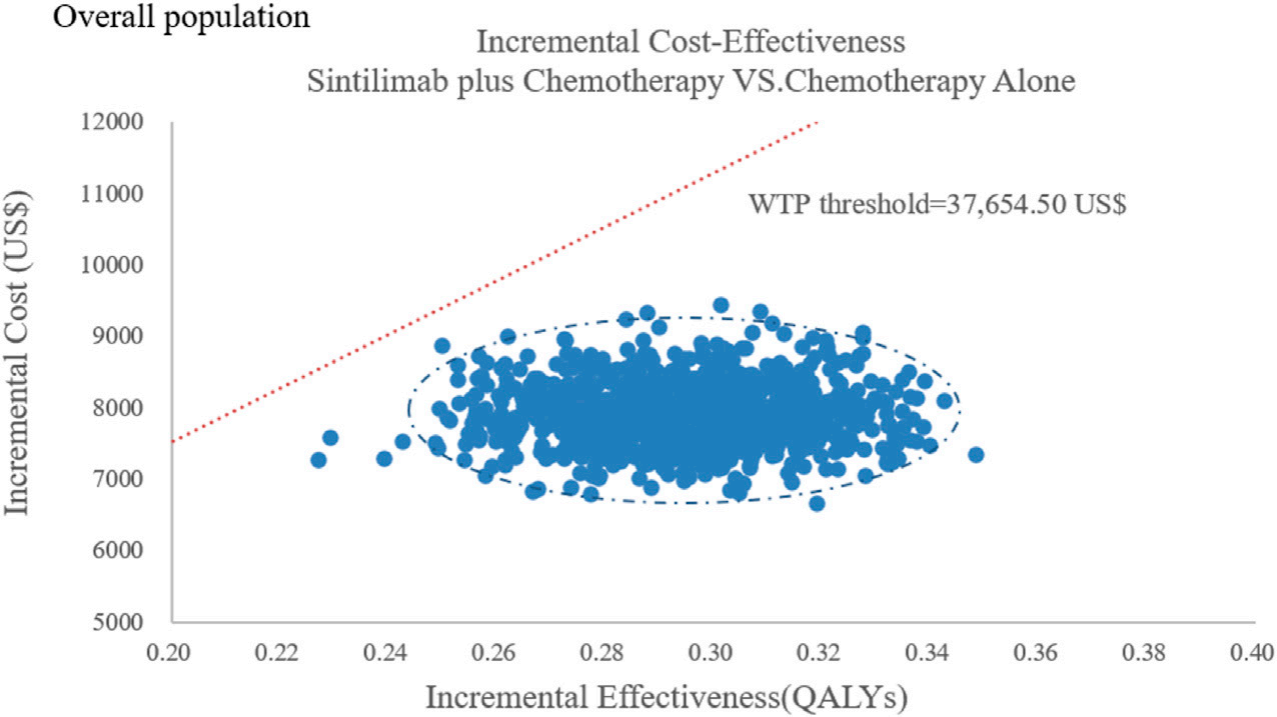
Probabilistic sensitivity analyses in overall population.

**FIGURE 7:**
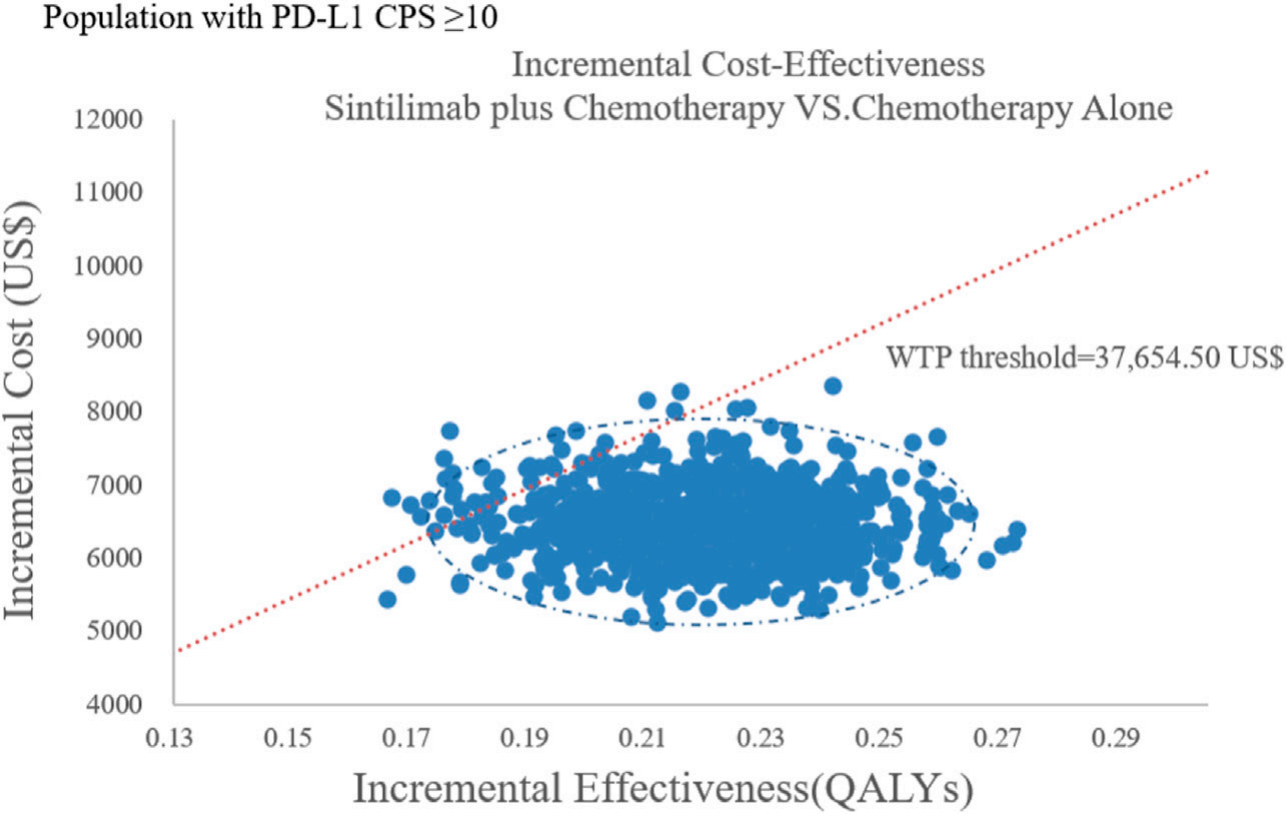
Probabilistic sensitivity analyses in the subgroup. Note: the dot represents the result of the Monte Carlo simulation. The diagonal line represents the WTP value, and the dot falls below the diagonal line to indicate that the test group has a cost effect compared with the control group.

The results of the probabilistic sensitivity analysis were summarized as a scatterplot and a cost-effectiveness acceptability curve. For sintilimab plus chemotherapy versus chemotherapy alone, about all of the PSA iterations were under the WTP threshold both in all population and the subgroup Figures 6, 7, which indicated that sintilimab plus chemotherapy was a cost-effective choice, compared with chemotherapy-alone treatment.

**FIGURE 8:**
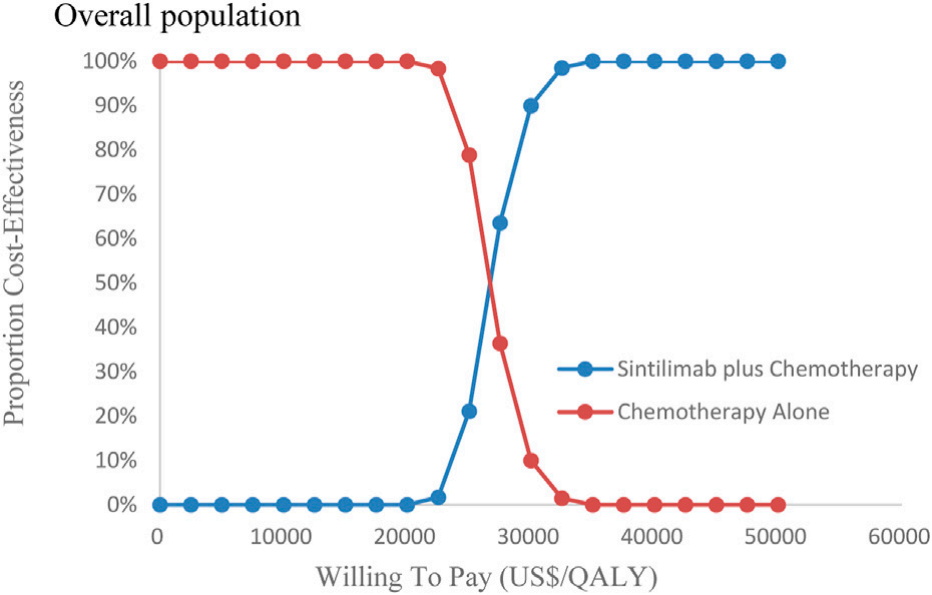
Cost-effectiveness acceptability curves in overall population (CEAC).

**FIGURE 9:**
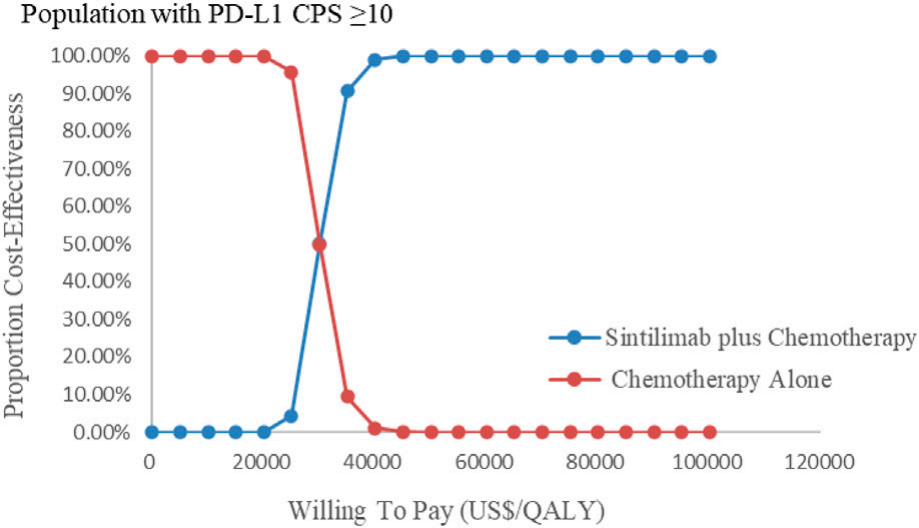
Cost-effectiveness acceptability curves in the subgroup (CEAC). Note: CEAC is a curve used to indicate the probability of a drug being economical. The magnitude of the WTP value directly affects the cost-effectiveness of the protocol. The acceptable curve shows the percentage of the cost-effectiveness of the simulation by using different treatment options, that is, the function of the relative change in the cost effect is the ICER threshold change.

Figures 8, 9 showed the cost-effectiveness acceptability curve from PSA. Compared with the chemotherapy-alone treatment, sintilimab plus chemotherapy exhibited a cost-effectiveness probability of 100% on the cost-effectiveness acceptability curve generated when the threshold was US$ 35,000 and US$ 40,000 in overall population and population with PD-L1 CPS ≥10, respectively. If the WTP threshold increased to 26,500 US$/QALY and 30,000 US$/QALY, sintilimab plus chemotherapy had a 50% chance to be cost-effective in overall population and population with PD-L1 CPS ≥10, respectively.

## Discussion

This study addresses the unmet need for an economic assessment of sintilimab plus chemotherapy. Based on the results of the ORIENT-15 trial, our analysis showed that sintilimab plus chemotherapy for locally advanced or metastatic esophageal squamous cell carcinoma was favorable at the willingness-to-pay thresholds of 37,654.50 US$/QALY. This finding is generally robust, as shown by the results of the probabilistic sensitivity analysis.

A recent corresponding economic evaluation reported the economic outcomes in China of using camrelizumab as a second-line regimen for advanced-stage esophageal squamous cell carcinoma, with an ICER of 86,745 US$/QALY (Yang et al., 2021). Another present analysis suggested that the camrelizumab plus chemotherapy might not be cost-effective as well, compared with chemotherapy alone (Zhang et al., 2021). Both studies demonstrated the cost-effectiveness of camrelizumab. As for other immune checkpoint inhibitors (ICI), nivolumab, as a second-line treatment for advanced esophageal squamous cell carcinoma, was also investigated and was not a cost-effective treatment option compared with chemotherapy (Zhang et al., 2020). In addition, a first-line regimen of pembrolizumab plus 5-fluorouracil and cisplatin for esophageal cancer therapy may not be as cost-effective as 5-fluorouracil and cisplatin, producing ICER values of $550,211/QALY in the United States and $244,580/QALY in China (Zhu et al., 2022).

One major strength of our analysis is the use of recently published 3-year survival data on the ORIENT-15 trials and the fact that the sintilimab plus chemotherapy and chemotherapy arms had been directly compared in the trial. Another strength is that almost 97% patients who enrolled in ORIENT-15 trials were from China, and only 19 of 659 patients were from outside of China. Hence, the trial represented the real-world data for Chinese patients with esophageal squamous cell carcinoma.

There are several limitations that need to be noted in this study. First, since the ORIENT-15 trial did not publish QoL data, information on the utility of PFS and PD was retrieved from the literature, which may have led to the deviations in the modeled results. Additionally, an incomplete follow-up from the trial data required extrapolation, which injects additional uncertainty about long-term outcomes. This is particularly true in the case of PFS curves of sintilimab plus chemotherapy in overall population as there were a limited number of events at the time of this data cut. However, we did follow the best practices for extrapolating survival data by using the best distribution fitted based on AIC and BIC criteria and visual inspections, as well as clinical plausibility. Third, we only considered the disutility of grade 1 and 2 AEs and not the cost; thus, we may have underestimated or overestimated the advantages of the treatment. Lastly, the strict limitations to the RCT may impose some limitations on the generalizability of the results of the pharmaco-economic evaluation typically. However, the ORIENT-15 study has fewer restrictions on inclusion criteria for the target population and did not strictly control the treatment regimen of the all patients, which is to meet the need for different clinical treatment options in different real-world settings. Therefore, it is feasible to perform cost-effectiveness analysis based on the data derived from the ORIENT-15 study (Lu et al., 2022).

## Conclusion

We established a model to estimate the cost-effectiveness of sintilimab plus chemotherapy for patients in locally advanced or metastatic esophageal squamous cell carcinoma. From our findings, we found that sintilimab plus chemotherapy was more cost-effective than chemotherapy alone for individuals with advanced esophageal cancer from the perspective of the Chinese healthcare system, regardless of expression of PD-L1.

## Data Availability

The original contributions presented in the study are included in the article/Supplementary Material; further inquiries can be directed to the corresponding authors.

## Abbreviations

QALYs, quality-adjusted life-years; ICER, incremental cost-effectiveness ratio; PSA, probabilistic sensitivity analysis; PFS, progression-free survival; OS, overall survival; PS, progression survival; KM, Kaplan–Meier; AIC, Akaike information criterion; AEs, adverse events; BSC, best supportive care; CT, computerized tomography; LYs, life-years; WTP, willingness-to-pay; GDP, capita gross domestic product; CPS, combined positive scores; BIC, Bayesian information criterion.
