# Supplementary material for: First-line sintilimab plus chemotherapy in locally advanced or metastatic esophageal squamous cell carcinoma: A cost-effectiveness analysis from China
Source: Front Pharmacol. 2022 Dec 7;13:967182. doi: 10.3389/fphar.2022.967182 (PMC9767976; doi:10.3389/fphar.2022.967182)
Supplement: Supplementary file 1 [file DataSheet1.ZIP › data_sheets/Table 1.docx]

Table 4 Key inputs for the cost-effectiveness model

| Parameter | value | low | high | distribution | source |
| --- | --- | --- | --- | --- | --- |
| Costs (US$) |  |  |  |  |  |
| Sintilimab/1000mg | 167.40 | 133.92 | 200.88 | Gamma | Negotiation price |
| Cisplatin/50mg | 11.78 | 9.42 | 14.14 | Gamma | Local charge |
| Paclitaxel/30mg | 11.44 | 9.15 | 13.73 | Gamma | Local charge |
| 5-fluorouracil/250mg | 9.61 | 7.69 | 11.53 | Gamma | Local charge |
| Docetaxel/20mg | 27.90 | 22.32 | 33.48 | Gamma | Local charge |
| Cost of BSC | 117 | 32.3 | 322.6 | Gamma | [15] |
| Cost of follow-up | 52.80 | 36.96 | 68.64 | Gamma | [20] |
| Chest enhanced CT | 134.37 | 107.50 | 161.24 | Gamma | [20] |
| Abdominal enhanced CT | 134.37 | 107.50 | 161.24 | Gamma | [21] |
| Cost of laboratory test | 90.32 | 72.26 | 108.38 | Gamma | [22] |
| Other costs |  |  |  |  |  |
| anti-tumor drug dispensing fees | 4.01 |  |  |  |  |
| Cost of IV | 2.78 |  |  |  |  |
| Hospitalization expense/day | 19.86 |  |  |  |  |
| Cost of SAEs |  |  |  |  |  |
| Anaemia | 669.45 | 535.56 | 803.34 | Gamma | [21] |
| Decrease in white blood cell count | 206.72 | 165.376 | 248.06 | Gamma | [21] |
| Decrease in neutrophil count | 544.19 | 435.35 | 613.43 | Gamma | [21] |
| Asthenia | 115.00 | 92.00 | 138.00 | Gamma | [23] |
| Increase in blood pressure | 1.35 | 1.08 | 1.62 | Gamma | [24] |
| Utility |  |  |  |  |  |
| PFS | 0.741 | 0.593 | 0.889 | Beta | [15,16] |
| PD | 0.581 | 0.465 | 0.697 | Beta | [15,16] |
| disutility due to grade 1 and 2 AEs | 0.01 | 0.008 | 0.02 | Beta | [17,18] |
| disutility due to grade 3 and 4 AEs | 0.16 | 0.11 | 0.204 | Beta | [17,18] |
| Accounting rate | 5% | 0% | 8% | Fix |  |

Abbreciation: BSC, best support care;SAEs, serious adverse events (≥grade 3) IV, intravenous injection; PFS, progression-free survival; PD, progression disease
