# Supplementary material for: First-line sintilimab plus chemotherapy in locally advanced or metastatic esophageal squamous cell carcinoma: A cost-effectiveness analysis from China
Source: Front Pharmacol. 2022 Dec 7;13:967182. doi: 10.3389/fphar.2022.967182 (PMC9767976; doi:10.3389/fphar.2022.967182)
Supplement: Supplementary file 1 [file DataSheet1.ZIP › data_sheets/Table 2.docx]

Table 5 Results for the cost utility analysis

|  | Total cost  (US$) | LYs | QALYs | Δ Costs  (US$) | ΔQALYs | ICER  (US$/QALY) |
| --- | --- | --- | --- | --- | --- | --- |
| Overall population | |  |  |  |  |  |
| Sintilimab plus chemotherapy | 15,399.21 | 1.44 | 0.90 | 7,923.63 | 0.30 | 26,773.68 |
| Chemotherapy alone | 7,475.58 | 1.04 | 0.61 | NA | NA | NA |
| Population with PD-L1 CPS≥10 | | |  |  |  |  |
| Sintilimab plus chemotherapy | 15,656.19 | 1.39 | 0.89 | 6,493.43 | 0.22 | 30,065.50 |
| Chemotherapy alone | 9,162.77 | 1.08 | 0.68 | NA | NA | NA |

Abbreciation: QALYs: quality-adjusted life-years; ICER: incremental cost-effectiveness ratio, NA, Not A Note: Δ: incremental
