# Supplementary material for: First-line sintilimab plus chemotherapy in locally advanced or metastatic esophageal squamous cell carcinoma: A cost-effectiveness analysis from China
Source: Front Pharmacol. 2022 Dec 7;13:967182. doi: 10.3389/fphar.2022.967182 (PMC9767976; doi:10.3389/fphar.2022.967182)
Supplement: Supplementary file 2 [file DataSheet2.ZIP › data_sheets/Supplemental Materials.docx]

**First-line sintilimab plus chemotherapy in locally advanced or metastatic oesophageal squamous cell carcinoma: a cost-effectiveness analysis from China**

1:baseline characteristics of the patients

|  | Sintilimab plus chemotherapy | Standard chemotherapy |
| --- | --- | --- |
| Age (years): |  |  |
| Median (IQR) | 63 (57-67) | 63 (57-67) |
| <65 | 189 (58%) | 202 (61%) |
| ≥65 | 138 (42%) | 130 (39%) |
| Gender: |  |  |
| Men | 279 (85%) | 288 (87%) |
| Women | 48 (15%) | 44 (13%) |
| Race: |  |  |
| Asian | 320 (98%) | 321 (97%) |
| White | 4 (1%) | 8 (2%) |
| Not reported | 3 (1%) | 3 (1%) |
| Disease status at enrolment: |  |  |
| Metastatic | 285 (87%) | 287 (86%) |
| Local advanced | 42 (13%) | 45 (14%) |
| Chemotherapy regimen: |  |  |
| Cisplatin plus paclitaxel | 307 (94%) | 309 (93%) |
| Cisplatin plus 5-fluorouracil | 20 (6%) | 23 (7%) |
| PD-L1 expression (CPS): |  |  |
| CPS <10 | 139 (43%) | 139 (42%) |
| CPS ≥10 | 188 (57%) | 193 (58%) |

Abbreviation: IQR, interquartile range; CPS, combined positive score

Table 2 the value of AIC and BIC

| Types of distribution | Sintilimab plus chemotherapy OS | | Sintilimab plus chemotherapy PFS | | Standard chemotherapy OS | | Standard chemotherapy PFS | |
| --- | --- | --- | --- | --- | --- | --- | --- | --- |
|  | AIC | BIC | AIC | BIC | AIC | BIC | AIC | BIC |
| in all patients | |  |  |  |  |  |  |  |
| Exponential | 649.7547 | 653.5446 | 726.1925 | 729.9825 | 758.885 | 762.6901 | 752.2067 | 756.0119 |
| Weibull | 615.6429 | 623.2228 | 698.0775 | 705.6574 | 718.0952 | 725.7055 | 692.5922 | 700.2025 |
| Gompertz | 631.4136 | 638.9936 | 720.9019 | 728.4818 | 735.6034 | 743.2137 | 725.2064 | 732.8166 |
| Log-normal | 613.2866 | 620.8665 | 672.4213 | 680.0012 | 714.9304 | 722.5407 | 679.4262 | 687.0364 |
| Log-logistic | 610.3871 | 617.967 | 675.2949 | 682.8749 | 713.626 | 721.2362 | 680.4558 | 688.0661 |
| Gen-gamma | 613.2811 | 624.651 | 673.661 | 685.0308 | 714.4419 | 725.8573 | 679.9881 | 691.4035 |
| in patients with CPS≥10 | |  |  |  |  |  |  |  |
| Exponential | 359.0264 | 362.2628 | 498.8872 | 502.1236 | 430.3089 | 433.5716 | 425.3247 | 428.5874 |
| Weibull | 331.9929 | 338.4658 | 487.4581 | 493.931 | 407.0693 | 413.5947 | 388.1698 | 394.6952 |
| Gompertz | 340.1515 | 346.6244 | 498.7202 | 505.1931 | 416.9549 | 423.4803 | 405.877 | 412.4023 |
| Log-normal | 333.4613 | 339.9342 | 468.1432 | 474.6161 | 403.9922 | 410.5176 | 382.7506 | 389.276 |
| Log-logistic | 330.6123 | 337.0852 | 471.3219 | 477.7948 | 404.7849 | 411.3103 | 382.408 | 388.9334 |
| Gen-gamma | 333.0212 | 342.7305 | 469.1594 | 478.8687 | 405.4148 | 415.2029 | 383.6583 | 393.4464 |

Abbreviation: OS, overall survival; PFS, progression-free survival; AIC, Akaike information criterion; BIC, Bayesian information criterion; CPS≥10, combined positive scores of ≥10

3: the shape parameter and scale parameter

| OS and PFS  curves | Optimal fitting  distribution | Shape parameter | Scale parameter |
| --- | --- | --- | --- |
| In overall population |  |  |  |
| OS curves of Sintilimab  plus chemotherapy | Log-logistic | 2.828918 | 0.5166103 |
| PFS curves of Sintilimab plus chemotherapy | Lognormal | 2.1923 | 0.8939621 |
| OS curves of chemotherapy | Log-logistic | 2.512269 | 0.513398 |
| PFS curves of chemotherapy | Lognormal | 1.733157 | 0.7921379 |
| In patients with CPS≥10 |  |  |  |
| OS curves of Sintilimab  plus chemotherapy | Log-logistic | 2.862995 | 0.4700579 |
| PFS curves of Sintilimab plus chemotherapy | Lognormal | 2.270911 | 0.9233276 |
| OS curves of chemotherapy | Lognormal | 2.583066 | 0.8959587 |
| PFS curves of chemotherapy | Log-logistic | 1.800978 | 0.4471674 |

Abbreviation: OS, overall survival; PFS, progression-free survival.
